# Medical Schools of the West

**Published:** 1842-06

**Authors:** 


### MEDICAL SCHOOLS OF THE WEST.

Next winter, there will be seven Medical schools in operation in the Valley of the Mississippi and the Lakes. They belong to four States, as follows: In Kentucky—the Medical Department of Transylvania University, and Medical Institute of Louisville; in Ohio, the Medical College of Ohio, and Willoughby University of Lake Erie; in Missouri, the Medical Department of Kemper College and the Medical Department of the University of St. Louis; in New Orleans, the Medical College of Louisiana.

In the Union there are 25 schools, or one for every 682,746 inhabitants; the population of the "Old Thirteen," including Florida, is 10,685,594, and its schools 18 in number; it has, then, one for every

593,644; the new States contain 6,376,972, which divided by seven, gives one school for every 910,996. It is obvious, then, that while seven sounds large for the Valley of the Mississippi and the Lakes, it is decidedly less than eighteen for the old States. To reject fractions we may say, that while the new have one medical institution for every 910 thousand inhabitants, the old States have one for every 593 thousand. Eleven schools in the Valley would not, then, be as large a proportion as now exists in the Atlantic basin. We must confess, that this result has rather taken us by surprise, for we had, to some extent, fallen into the common opinion, that the number of western schools is excessive. And excessive it may be, considered in reference to sound policy, but it is far from being so, compared with the number in the mother States; and as they are nearly stationary, while the daughters are rapidly increasing in population, if retrenchment is to be made any where, it should be among them. We are not prepared, however, to propose retrenchment even there, not being convinced, that the number of schools is too great for the Union, but rather that the number of *their* pupils is too few. *Nota bene* that we have underscored "their," for the entire number of students in the United States is *not* too great. It is only, that too few of them attend lectures. If all who engage in the study of medicine were compelled to graduate, our 25 schools would be filled, for there are at all times even more than 5000 engaged in the study; but instead of that number, not more than 2500 seek the benefits of university instruction. Let the other 1500 come forward to qualify themselves in a proper manner, and our schools will average 200 each—as large a number as they ought to contain. D.

